# Spatiotemporal Mechanisms of the Coexistence of Reintroduced Scimitar-Horned Oryx and Native Dorcas Gazelle in Sidi Toui National Park, Tunisia

**DOI:** 10.3390/ani14101475

**Published:** 2024-05-15

**Authors:** Marouane Louhichi, Touhami Khorchani, Marie Petretto, Douglas Eifler, Maria Eifler, Kamel Dadi, Ali Zaidi, Yamna Karssene, Mohsen Chammem

**Affiliations:** 1 Laboratoire d’Elevage et de Faune Sauvage, Institut des Régions Arides (IRA), Medenine 4119, Tunisia; marouane.louhichi@gmail.com (M.L.); touha2009@gmail.com (T.K.); alizaidi48@gmail.com (A.Z.); yamna_karssene@yahoo.fr (Y.K.); 2 Faculty of Sciences of Gabes, University of Gabes, Gabes 6072, Tunisia; 3 Marwell Wildlife, Colden Common, Winchester SO21 1JH, UK; mariep@marwell.org.uk; 4 Erell Institute, 2808 Meadow Drive, Lawrence, KS 66047, USA; doug.eifler@gmail.com (D.E.); maria.eifler@gmail.com (M.E.); 5 Laboratoire des Écosystèmes Pastoraux et Valorisation des Plantes Spontanées et des Microorganismes Associés, Institut des Régions Arides (IRA) de Medénine, Medenine 4119, Tunisia; dadi2022@yahoo.com

**Keywords:** activity patterns, antelopes, arid ecosystem, camera-trap, management and conservation, modelling habitat suitability, spatiotemporal niche

## Abstract

**Simple Summary:**

Conservation strategies often involve reintroducing species back into parts of their historic range where they no longer occur. Reintroduction efforts can be complicated when there are native species present that might compete with the reintroduced species. Exploring the relationship between the existing and reintroduced species can improve the success of reintroduction efforts. Using information from camera-traps, we examined the extent to which reintroduced oryx and native gazelle display similar activity and space use patterns in Sidi Toui National Park, Tunisia. The two species exhibited minimal spatial overlap within the park and favoured habitats with different vegetation features. Activity patterns relative to time of day and season were similar for oryx and gazelle. Both antelope species were most active at dawn and dusk. Seasonally, activity was lowest for both when conditions were hot and dry and was highest following the rainy season in spring, when new vegetation emerged. The differences in space use patterns can facilitate coexistence between the two species of grazers in Sidi Toui National Park. Habitat diversity can be a key determinant for allowing reintroduced and native species to coexist.

**Abstract:**

Examining the distribution patterns and spatiotemporal niche overlap of sympatric species is crucial for understanding core concepts in community ecology and for the effective management of multi-species habitats within shared landscapes. Using data from 26 camera-traps, recorded over two years (December 2020–November 2022), in Sidi Toui National Park (STNP), Tunisia, we investigate habitat use and activity patterns of the scimitar-horned oryx (*n* = 1865 captures) and dorcas gazelle (*n* = 1208 captures). Using information theory and multi-model inference methods, along with the Pianka index, we evaluated the habitat characteristics influencing species distribution and their spatial niche overlap. To delineate daily activity patterns, we applied kernel density estimation. Our findings indicate minimal spatial overlap and distinct environmental factors determining suitable habitats for each species. Furthermore, we found significant temporal niche overlaps, indicative of synchrony in daily activity patterns, with both species showing peak activity at dawn and dusk. Our results indicated that oryx and gazelle differ in at least one dimension of their ecological niche at the current density levels, which contributes to their long-term and stable coexistence in STNP.

## 1. Introduction

In conservation biology, maintaining and restoring biodiverse ecosystems is essential, especially in arid zones where resources are scarce and conditions are harsh [1]. Wild ungulates serve as crucial ecological indicators of ecosystem health, impacting ecosystem structure and function [2]. They are an intricate component of grassland food webs, exerting significant direct and indirect effects. Activities that include grazing, browsing, trampling, and defecation can reshape plant communities and influence nutrient cycles [3]. However, ungulates face a variety of threats across the globe that are significantly affecting their populations. Threats vary by region and species but typically include habitat loss [4], poaching [5], climate change [6], and competition with livestock [7]. Moreover, ungulates can affect the responses of associated animals to the ecosystem [8,9].

The reintroduction of animal species through the release of either wild or captive-bred individuals, is potentially valuable to conservation programs aiming to re-establish species in their historic ranges, following extinction or disappearance [10]. Sidi Toui National Park (STNP) in Tunisia is among the sites privileged to be chosen for the reintroduction of the scimitar-horned oryx (*Oryx dammah*), hereafter referred to as oryx. The last recorded sightings of oryx in the wild occurred towards the end of the 1980s [11]. The species was declared extinct in the wild in 1999 [12]. However, in the same year, 10 individuals coming from European zoos have been released in STNP [13], supplemented by two individuals translocated from Bou Hedma National Park, Tunisia in 2009. Approximately 70–80 oryx were regularly counted during the study period (2020–2022). These additions further compounded the ecological dynamics, as they represent potential ecological equivalents and competitors of the dorcas gazelle (*Gazella dorcas*), hereafter referred to as gazelle, a vulnerable yet symbolic indigenous species in STNP [14]. Gazelles are not counted in STNP; however, small groups of gazelles are commonly observed during the transects and the park staff estimate their number to be around 80 individuals. In December 2023, after 24 years of the world’s most ambitious reintroduction program, the oryx has returned to the wild and has been reclassified as ‘Endangered’ by the International Union for the Conservation of Nature (IUCN) Red List, having formerly been categorized as ‘Extinct in the Wild’ [15].

The reintroduction of large herbivores can be followed by rapid population growth, often due to low large predator populations and controlled hunting, which can lead to competition with native species [16,17,18]. When coexisting species have similar resource requirements, competitive exclusion can occur [19,20], or they can exert strong negative effects on each other [21]. Interspecific competition significantly shapes the ecological and demographic dynamics of coexisting species through resource exploitation and interference interactions [22,23], which can become manifest across the fundamental niche dimensions of space, time, resources, and predators [24]. Ecologically similar species can coexist by differing morphologically when resources are limited, or behaviourally through niche differentiation [25,26], by making use of different resources or using the same resources at different spatial or temporal scales [27,28].

Understanding how coexisting species differentiate their niches is beneficial for the conservation and management of healthy ecological communities [29,30]. The temporal partitioning of niches can reduce agonistic or competitive encounters [31]. Ecologically similar herbivores can also reduce competition through large scale spatial segregation [32], while, at a fine scale, coexistence is facilitated by the selection of different forage plants [33,34], particularly when body size [35] or foraging behaviour differs among ungulate species [36].

Examining daily activity patterns can provide insight into how sympatric species that share resources partition time and space to promote stable coexistence [37,38]. Activity patterns vary significantly across regions and seasons, influenced by factors like day length, moonlight [39], and interactions related to competition or predation [40,41]. Activity patterns can be assessed through direct observation [42] or by using activity loggers, GPS, or VHF collars, which involve extensive surveying, capture efforts, and equipping of animals [43], which can have negative impacts on animal populations, as well as limitations in challenging terrain and dense vegetation [44]. Recent studies have highlighted the effectiveness of camera-trap methods for assessing the activity of target species and their interspecific temporal and spatial overlap [45,46,47]. Camera-traps are favoured in wildlife research for their non-invasiveness, cost-effectiveness, affordability, and ability to provide extensive datasets on species activity [48,49]. The time-stamped data they generate can provide valuable insights into potential interactions and activity periods, which have practical applications for wildlife conservation and management [50]. Camera-traps have been used to obtain detailed activity patterns of various wild ungulates [47,51,52,53,54,55].

We aim to evaluate the effect of reintroduced oryx in STNP on the ecosystem, particularly the coexistence between oryx and gazelle. Most behavioural studies of wild ungulates have focused on single species ecology, few have explored spatial and temporal activity patterns of wild ungulates across multiple seasons [56,57]; limited research has examined interspecies interactions [47], potentially leading to inconsistent conservation strategies. We use camera-traps to test the hypothesis that spatiotemporal niche segregation facilitates the coexistence of gazelle and oryx in STNP. With the aim of ecosystem restoration, while accounting for potential impacts on native species, we examine daily and seasonal activity for each species, model seasonal habitat suitability, and quantify the spatiotemporal niche overlap between the two species. Our findings can be used to inform future reintroductions and to help assess the effectiveness of conservation programs aimed at restoring the original ecosystem, while considering potential impacts on native species.

## 2. Materials and Methods

### 2.1. Study Area

**Figure 1:**
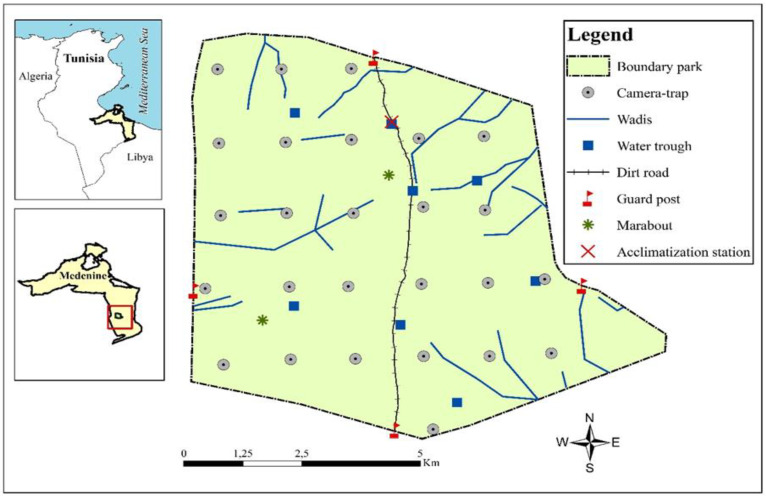
Localization and delimitation of STNP and the placement of camera-trap stations.

Data were collected throughout STNP on the periphery of the Sahara Desert, near the Tunisian–Libyan border (Figure 1), 54 km south of Ben Gardane, Medenine governorate, southeastern Tunisia (11.24° E, 32.70° N). The park contains 6315 ha, with a maximum elevation of 178 m asl, occurring on the boundary between the upper Saharan temperate zone and the lower arid cool zone, characterized by low and sporadic rainfall, with an average annual precipitation range of 100–125 mm. Summers are dry and scorching, with temperatures often soaring to ca. 45 °C [58]. The park encompasses Djebel Sidi Toui, a hill encircled by a vast plain consisting of small dunes, sebkhas, and dry sandy wadis, serving as a sanctuary for a cluster of 14 ancient religious sites (Marabouts). STNP is dominated by steppe vegetation, including grasses (e.g., *Cenchrus ciliaris*, *Stipa lagascae*, and *Stipa retorta*), forbs (e.g., *Atractylis serratuloides*, *Diplotaxis harra*, and *Medicago minima*), and shrubs (e.g., *Argyrolobium uniflorum*, *Helianthemum sessiliflorum*, and *Ziziphus lotus*) [59]. The park’s vertebrate fauna is diverse, hosting various Saharan protected mammals such as *Vulpes zerda* (Fennec fox), *Canis anthus* (African golden wolf), *Vulpes vulpes* (Red fox), and Sahelo–Saharan bovids including *Oryx dammah* (oryx) and *Gazella dorcas* (gazelle). Additionally, STNP contains a wealth of avian fauna that includes non-migratory species (i.e., *Alectoris barbara* (Barbary partridge), *Pterocles alchata* (pin-tailed sandgrouse), *Alauda arvensis* (Eurasian skylark), *Corvus corax* (common raven), and *Cursorius cursor* (cream-colored courser), as well as numerous migrants, due to the park’s location along the Mediterranean and trans-Saharan migration paths. Finally, there are some rare and protected reptiles (i.e., *Chamaeleo chamaeleon* (common chameleon), *Uromastyx acanthinura* (North African spiny-tailed lizard), and various snake species).

### 2.2. Data Collection

#### 2.2.1. Camera-Trap Survey

We used data from 26 camera-traps (Bushnell Trophy Cam HD Aggressor; Bushnell Outdoor Products, Overland Park, KS, USA) installed in STNP. The cameras were distributed across the study site, according to a grid design developed in QGIS (QGIS Geographic Information System, 2018). Each camera was placed as close as possible to the centre of the grid cell, ca. 1.5 km apart, to comprehensively cover the study area (Figure 1). To maximise detection rates, the cameras were affixed to rocks at heights ranging from 40 to 50 cm above the ground, with their lenses oriented towards animal tracks or open areas, to capture medium- to large-bodied animals (>1 kg) [60]. All camera-traps were set to take a series of three photos at the highest image quality, when triggered (File S1). Intervals between triggers were set at the lowest value permitted by the camera model (0.6–5 s).

The cameras were deployed on 21 October 2020. Following their installation, the cameras underwent an initial check 24 h later and, subsequently, approximately once per month. These regular checks aimed to minimize disruption at the sampling site and to ensure the proper functioning and positioning of the devices. During each inspection, the memory cards were replaced with empty ones and the contents were later downloaded for analysis. Supervised by the Marwell Wildlife’s team, two park guardians conducted monthly monitoring of the cameras. Their duties included verifying the position of each camera, ensuring battery functionality, and downloading captured images. Our approach in camera-trapping studies aligns with established methodologies used in similar research conducted in Southern Tunisia, including STNP [55,61,62]. These studies provide evidence of successful deployments and data collection techniques in arid habitats, contributing to our methodological framework.

#### 2.2.2. Data Processing

To mitigate the potential bias resulting from the novelty effect [63], we did not use photos taken during the first two months following camera installation (i.e., October and November 2020), evaluating camera-trap data obtained from December 2020 to November 2022. Seasonally, we considered fall to include September—November, winter to include December–February, spring to include March–May, and summer to include June–August [64]. We assumed that any individual captured by a camera-trap was active [65]. We manually consolidated data from camera-traps into a database, summarizing each capture event by camera location, species photographed, date, and timestamp. When an image contained multiple species, each individual was treated as a separate, independent capture. To prevent pseudo-replication, we considered photos of the same individual, identified by distinctive horn shapes or unique markings, to be independent, if they were taken by the same camera and were separated by the commonly accepted standard in camera-trap studies of ≥30 min [40,48,66,67]. To evaluate periodicity in daily activity patterns, we converted time into radians (with 2π radians corresponding to a 24 h period) [65].

#### 2.2.3. Environmental Data

Ecological and environmental habitat variables that can influence the presence and behaviour of wildlife include type of vegetation, climate, topography, proximity to water sources, and levels of human disturbance [68,69]. First, we measured five variables to assess habitat suitability. We used the point-quadrat method [70] along line transects to categorize vegetation and to estimate the percent coverage of each category for each season within a 100 m radius of each camera-trap location. We randomly located 5–20 m transects at each camera site, lowering sampling pins every 20 cm (*n* = 100 total points per transect). Where pins intercepted vegetation, we recorded the vegetation category as either (1) shrub (SC), (2) forb (FC), or (3) grass (GC); summed the intercept measurements; and converted the sum into a percentage, to calculate total cover for the camera site location and for each vegetation category [59]. For analysis, we averaged the total cover within each season for each vegetation category across the five transects at each camera-trap location. We also computed the (4) distance from each camera to the closest wadi (WA) and recorded the (5) altitude (m above sea level) of each camera-trap (AL).

Second, using five additional variables, we assessed the levels of human disturbance by measuring the (1) distance from each camera to the closest of the seven guardhouses or marabouts (DP) that could potentially hinder the movement of oryx and gazelle (i.e., park entrances—Magroun, Mhijra, Madi, and Hawach; acclimatization station—Zriba; and major marabouts frequently visited by pilgrims—Rotila and Torki; Figure 1). Additionally, using the park infrastructure map, we measured the (2) distance between each camera-trap and the closest main dirt road travelled by passenger cars (DR) and the (3) nearest fence (Figure 1). Due to the fact that, in hot environments like southern Tunisia, ungulates rely heavily on water sources and shade for maintaining water balance, we also recorded GPS coordinates for and calculated the distance between each camera and the (4) nearest artificial water trough (*n* = 8; WT, Figure 1) and (5) shade shelter (*n* = 10).

### 2.3. Data Analysis

We used ArcGIS^®^ (Version 10.8, Environmental Systems Research Institute Inc., (Esri, Redlands, CA, USA, 2023) to map GPS locations for measures of human disturbance, wadis, water trough, and shade shelter, as well as to calculate their distance relative to camera-traps and camera-trap altitude (Figure 1).

#### 2.3.1. Relative Activity Index (RAI)

We calculated the relative activity index (RAI) using different methods, to identify spatial and temporal activity patterns. First, we used RAI1 to examine the spatial distribution of oryx and gazelle and to evaluate their respective prevalence or dominance between sites [71], as well as to discern the importance of environmental factors on their distribution patterns [72]. We used *t*-tests to statistically evaluate differences in RAI1 values for each species at each site and between the two species.

RAI1 = (total number of independent detections of each species/total number of camera days at each site) × 100

Second, we calculated RAI2 to assess the temporal activity patterns of each antelope [73]. To evaluate the statistical significance of differences in annual species detections, we utilized an Analysis of Variance (ANOVA) test.

RAI2 = (total number of independent detections for each species/total number of camera days within a specific month (*i*)) × 100 camera days

#### 2.3.2. Spatial Niche Analysis

##### Modelling Habitat Suitability

We applied information-theoretic and multi-model inference (MMI) techniques to the spatial RAI1 data of the two species in each camera-trap site, to predict the factors influencing the distribution of oryx and gazelle in different seasons [74]. To examine the relationship between the RAI1 of each species and the explanatory variables, we ran Generalized Linear Models (GLMs) analysis [75] using the Gaussian distribution. To avoid autocorrelation and multicollinearity, we used Spearman’s rho correlation coefficient to test for pairwise correlations among the predictor variables. We retained predictors with a correlation < 0.7 [76]. Because the fences were strongly correlated with the guardhouse post and marabout, and water troughs were correlated with wadis and man-made shade structures, we did not use fences and shade structures for analyses, but retained the remaining eight predictor variables.

We used the Akaike Information Criterion (AIC) [75] to compare alternative models, employing AICc to correct for small samples (n/k < 40: n camera sites = 26 and k variables = 8 [77]. AICc was calculated for each model in the dataset and we considered the model with the lowest AICc value (AICcmin) as the best, indicating the most parsimonious fit. For a set of competing models, we used MMI to find the best fit [77], ranking candidate models by calculating the AICc differences (Δi) relative to AICcmin. A larger Δi indicates a weaker model, while Δi < 2 means that the models are not significantly different [77].

We assessed the relative importance of predictors in determining the habitat suitability for each species through the following two approaches: (1) the predictor selection probability, which represents the likelihood of a predictor being included in the top models, if the analysis were repeated with a different dataset [78]; and (2) the model-averaged coefficients, which indicate the magnitude of each predictor’s contribution to variation in the habitat suitability index. Additionally, we assessed the level of agreement between the best model in each season and the explanatory variables with adjusted R^2^, where R^2^ > 0.40 indicated an accurate model with strong predictive capabilities.

##### Spatial Niche Overlap

To assess annual and seasonal spatial overlap, we quantified the ecological niche overlap between the two species, using the Pianka Niche Overlap Index on the RAI1 values of each species at each camera site [79]. The index yields values ranging from 0 to 1, where 0 indicates no ecological niche overlap (i.e., the two species occupy entirely distinct ecological niches), while 1 indicates complete niche overlap (the two species occupy precisely the same ecological niche).

#### 2.3.3. Temporal Niche Analysis

##### Daily Activity Patterns

Recent analyses of diel activity patterns adopt graphical representations, utilizing nonparametric kernel density estimates (KDEs) derived from camera-trap data [40,65], which allows for a continuous depiction of activity over a 24 h cycle. Graphical KDEs display temporal variations in activity, including peak activity periods and behavioural categorizations related to daily routines. We classified the diel cycle based on local sunrise and sunset times [80], to examine whether the daily activity pattern of each species was primarily diurnal, nocturnal, or crepuscular. Crepuscular activity was defined by the 1 h interval before and after sunrise and sunset [81,82]. We obtained a date-adjusted sunlight hours calendar for STNP from the Ben Gardane city calendar (https://dateandtime.info/fr/citysunrisesunset.php?id=2472431, accessed on 3 November 2023). Subsequently, we calculated the average times of sunrise and sunset for each month.

To investigate whether activity patterns were predominantly crepuscular, diurnal, or nocturnal for each species, we computed selection ratios [83] using the following formula:

(1) wi=oi/πi

where *wi* represents the selection ratio for period *i*, *oi* is the proportion of detections in period *i*, and *πi* denotes the proportion of the length of period *i* relative to the total length of all periods. A selection ratio of *wi* > 1 indicated selective usage of the time period, while *wi* < 1 signified avoidance. We used analysis of variance (ANOVA) to assess whether activity patterns were non-random for each species.

##### Temporal Niche Overlap

Using the coefficient of overlap (Δ) [65], we examined annual and seasonal temporal niche overlap in the activity of oryx and gazelle. The coefficient Δ is defined as the area under the curve formed by taking the minimum of two kernel density functions at each point in time [84], varying from 0 (no overlap) to 1 (complete overlap). We used Δ_1_ when the smaller of two samples contained < 75 observations, and Δ_4_ when both samples contained ≥ 75 observations [40,85]. We categorized the strength of overlap in the activity patterns between the species as strong if Δ > 0.75, moderate if 0.5 ≤ Δ ≤ 0.75, and low if Δ < 0.5 [86]. Furthermore, we calculated 95% confidence intervals for each Δ_4_ value, using smoothed bootstrap estimates with 10,000 resamples [85].

We used R [87] for all analyses, examining activity patterns and spatiotemporal overlap using the “overlap” package [85] and the “MuMIn” package (1.47.5, March 2023) for MMI.

## 3. Results

### 3.1. Inventory Data

**Figure 2:**
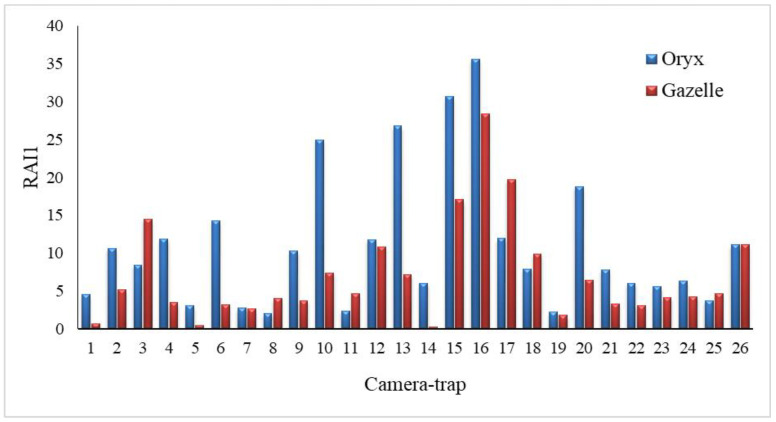
Relative activity index (RAI1) for each antelope species at each camera-trap (the total number of independent detections, divided by the total number of camera days, and multiplied by 100) throughout the study period in STNP.

We accumulated a total of 17,323 camera-trap days useable for data analyses, obtained from December 2020 to November 2022. We captured 10,938 independent detections of 11 different wildlife species, including 3073 of the two target ungulates (mean per camera-trap ± standard deviation = 118.19 ± 102.75). We detected our two focal species in all 26 camera-traps (Figure 2), with oryx accounting for most (60.69%) of the independent captures (*n* = 1865), whereas gazelle totalled 1208 captures (39.31%).

### 3.2. Relative Activity Index

We found significant spatial variation for each species across camera-trap sites in each season (*p* < 0.001). Despite the higher presence of oryx compared to gazelle in the majority of camera-trap sites (Figure 2), there were no significant differences between the RAI1 values for camera captures of the two species during the entire study (t = −0.784, df = 101, *p* = 0.435). Seasonally, only in the winter 2020–2021 (t = 2.745, df = 30, *p* = 0.010) and fall 2021 (t = 2.954, df = 37, *p* = 0.005) did we find significant differences between the species in RAI1 values for captures at camera sites.

**Figure 3:**
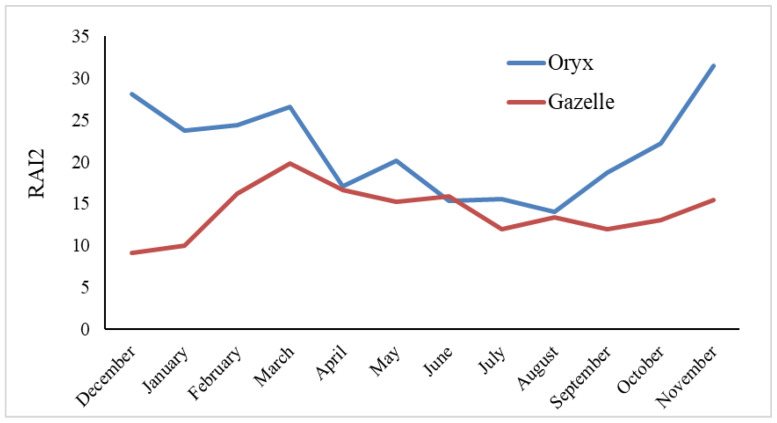
Relative activity indices (RAI2, or the number of detections per 100 camera days) for oryx and gazelle for every month in the STNP. RAI2 from each month of the two years were combined.

The oryx exhibited heightened activity in November, December, and March, while experiencing reduced activity between June and August (Figure 3). Annual variation in oryx activity was significantly non-random (F = 3.998, df = 3, *p* = 0.022). According to the post hoc test, seasonal variation in oryx activity arose from the difference between winter and summer (*p* = 0.023). Despite peak activity for gazelle occurring between February and April, particularly in March, and reduced activity during December and January, there was no significant seasonal variation in gazelle activity.

### 3.3. Spatial Niche

#### 3.3.1. Modelling a Suitable Habitat

When comparing the top habitat suitability models based on AICc for both oryx and gazelle across different seasons, no single model fit the data (ΔAICc < 2, Table 1 and Table 2). However, grasses emerged as the primary factor influencing oryx habitat selection and were present in all seasons with relatively high coefficients, except in the fall. During the winter and summer seasons, the most parsimonious models for oryx (Δi < 2) included only grasses as a significant predictor, with a high probability of selection (≥0.78). Forbs had a relatively smaller effect, but played an important role in fall and spring, contributed significantly to oryx spatial occupation, and were more important than grasses in spring (Table 1). The remaining variables exhibited low selection probabilities in all seasons (<0.56).

Regarding habitat suitability for gazelle, forbs exhibit the strongest coefficient and highest selection probability across seasons, except in summer (Table 2). The other variables had an effect over one or two seasons. Grasses were present with a moderate selection probability over two seasons, with a coefficient that was negative (−2.957) in winter 2020–2021 and positive (16.269) in spring 2022. Dirt road had a high probability of selection (≥0.88) in winter 2020–2021 and summer 2021, with negative coefficients (−0.001 and −0.004, respectively). Water troughs were only significant in spring 2022 (selection probability = 0.80). In summer 2021, altitude had a moderate effect with a negative coefficient (−0.396). Likewise, the distance from the guardhouse post and marabout had a moderate selection probability (0.67) in the winter of 2020–2021. The selection probability of wadi was relatively high (0.88) in summer 2022 (Table 2). Shrubs were not significant in any season.

#### 3.3.2. Spatial Niche Overlap

Throughout the entire study, oryx and gazelle showed relatively low spatial niche overlap (≤ 0.57; Table 3). The Pianka index for seasonal spatial niche overlap reached a minimum in winter 2020–2021 (0.31) and maximum in fall 2022 (0.57).

### 3.4. Temporal Niche

#### 3.4.1. Daily Activity Pattern Characteristics

**Figure 4:**
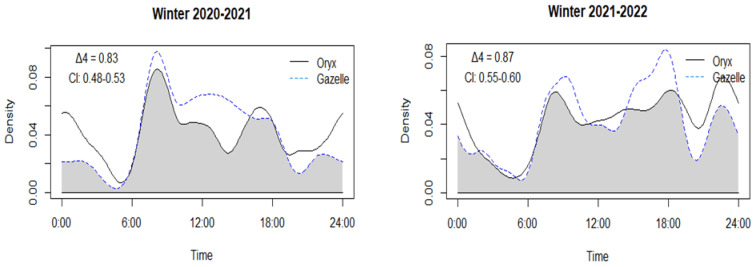

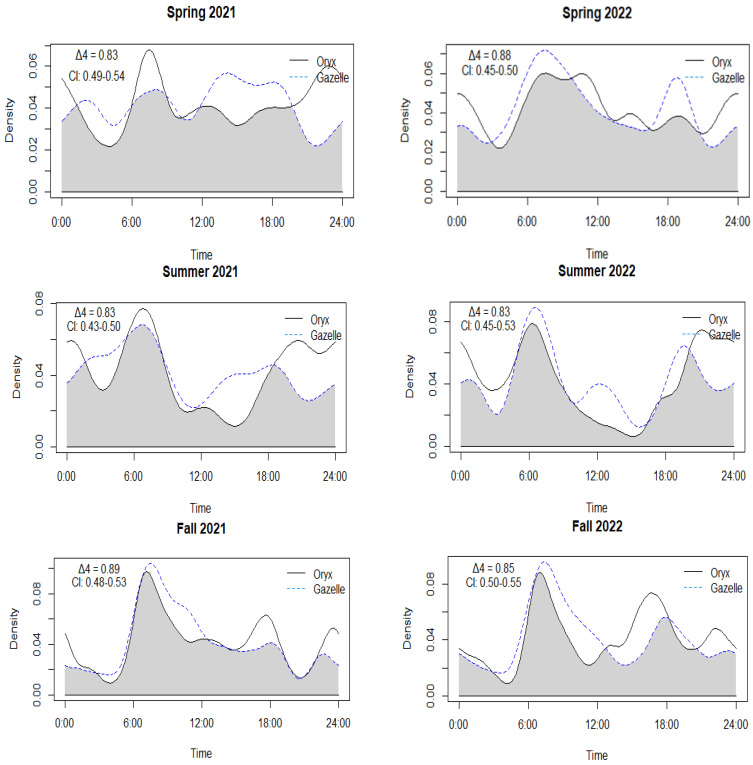
Diel activity patterns and overlap of oryx and gazelle in all seasons in STNP. The y–axis is the Kernel Density Estimates. The overlaps are denoted by the grey area. Δ_4_ is the overlap coefficient and CI is the 95% confidence intervals of overlap coefficient.

Generally, both antelope species were crepuscular, with a preference for dawn activity (Figure 4) and some variation in times seasonally. In fall and winter, oryx had bimodal activity peaks at dawn (06:00 h and 08:00 h) and dusk (16:00 h and 18:00 h), while in summer, oryx were mainly active at dawn (05:00 h and 07:00 h) and at night (21:00 h and 24:00 h). During spring of both years, oryx were active in the morning (06:00 h–08:00 h). Gazelle were more consistently active at dawn and dusk with bimodal peaks and, to a lesser extent, they were active during the day. Based on the selection ratio (*wi*; Table 4), both species were predominantly crepuscular and diurnal (*wi* > 1), usually having low levels of nocturnal activity (*wi* < 1), except for oryx in summer, when they were crepuscular and nocturnal. For gazelle, periods of activity patterns were non-random in most seasons (five of eight seasons), while, for oryx, the periods of activity were non-random during only two seasons (ANOVA, Table 4).

#### 3.4.2. Temporal Niche Overlap

Temporal niche overlap between oryx and gazelle remained consistently high during all seasons (Δ_4_ > 0.75, Figure 4). The seasonal temporal overlap values were all consistent, ranging from Δ_4_ = 0.83 to Δ_4_ = 0.89.

## 4. Discussion

Based on daily and seasonal activity patterns, our model of seasonal habitat suitability, and the spatiotemporal overlap between oryx and gazelle, our hypothesis that spatiotemporal segregation facilitates coexistence in STNP was partially supported. The daily activity rhythms of oryx and gazelle on the annual and seasonal time scales show high similarity and overlap (i.e., similar temporal niches). However, their annual and seasonal spatial niches have a low degree of overlap, indicating that the two species have niche separation under specific habitat conditions, which could be one of the mechanisms for oryx and gazelle to achieve a long-term, stable coexistence in STNP. Our findings are similar to the spatial and temporal patterns reported for oryx and gazelle at Dghoumes National Park in Tunisia [88,89].

### 4.1. Annual Spatial Niche Partitioning

The spatial distribution of the oryx within the park was more extensive than that of the gazelle (based on RAI1 results, Figure 2). A lower amount of captures of gazelle on camera-traps could be attributed to gazelle being camera-shy—they are highly vigilant [90] and, as part of their anti-predator (i.e., African golden wolf) strategy, engage in hiding behaviour [91], limited movement [92], or lower numbers. In addition, gazelle spend nearly 60% of their daily activity period at rest [93]. Oryx, in contrast, are characterized by long-distance movements following rainfall (including migration), in search of high-quality forage, particularly emerging annual plants and young green shoots [11,94]. Feeding behaviour can also influence the extent of the distribution area, especially when the oryx is defined as a grazer and the gazelle as a browser [95,96]. In addition, the larger body size of the oryx compared to the gazelle influences numerous biological processes [97], including the size and location of their foraging area [98]. Consequently, oryx require more time and a larger area to find suitable food sources to meet their energy needs, compared to gazelle.

In our model of habitat suitability (MMI), vegetation was the highest predictor of spatial niche use for both antelope species. Food of high quality and quantity is the primary determinant of resource partitioning among ungulates in savannah and arid environments [99], with food preferences playing a significant role in determining the spatial occupancy of the oryx and gazelle, potentially resulting in spatial niche separation. Oryx mainly favoured habitats in STNP that are rich in grasses (Poaceae; MMI). The diet of oryx is composed mainly of grasses [11,93,100,101], the presence of which strongly influences their spatial distribution [102,103].

Large herbivores tend to opt for taller grasses to meet their higher biomass dietary requirements, which often consist of a substantial amount of high-fibre material [104]. Low stomatal conductance and efficient CO₂ uptake contribute to adept water utilization by grasses. In addition to extracting water from the soil [105], tall grasses capture dew and fog, making them an attractive food source in arid lands, especially during the dry season [100]. Oryx meet their water needs by grazing on available vegetation [106] and can survive extended periods without drinking [102]. Small-bodied herbivores such as gazelle have lower absolute intake requirements than larger-bodied herbivores, enabling them to meet their intake needs on short, high-quality forage [107]. Indeed, gazelle in STNP preferred habitats rich in high-quality forage, such as forbs, which can account for 70% to 90% of their diet [108], rather than fibrous forage such as grasses [109].

### 4.2. Annual Temporal Niche Partitioning

The annual activity for oryx can be split into two main periods. The first period occurs after the rainy season (October–March), when oryx display high activity levels (based on RAI2 results, Figure 3). During the rainy season, the study region experiences its highest average rainfall (e.g., 17.2 mm (October)—ca. 19.5 mm (November and December)). The corresponding increased activity of oryx could be due to the emergence of new annual plants and buds following heavy rain. The second period of activity, during which oryx display reduced activity, occurs before the rainy season (April–September). Activity is particularly low when the average temperature is highest (i.e., summer—29 °C in July, 30 °C in August). To cope with high temperatures and low rainfall, oryx optimize water intake and minimize body water loss [110], most effectively by reducing movement and ingesting lower-quality food that requires longer digestion [111]. Moreover, the installation of shade structures near water points provided shade in addition to water, which, in combination, could account for the shift of oryx towards habitats with water sources [101]. Arid-land ungulates conserve water by adjusting their blood profile [112,113], which helps them to maintain consistent seasonal activity [92]. Even the daily activity patterns of oryx and gazelle were similar and temporally aligned (kernel density estimation analysis), which could account for their high temporal overlap during all seasons.

Generally, and consistent with many wild ungulates [46,47,114], oryx and gazelle were crepuscular, exhibiting bimodal activity peaks at dawn and dusk [115]. The primary environmental factor influencing the daily activity patterns of wild ungulates is solar radiation during sunrise and sunset [112,116], in response to photoperiod [43]. Both antelope species displayed their highest activity levels when temperatures were relatively mild and humidity was low. During the hottest part of the day, ungulates often seek refuge under trees to lower their body temperatures, resulting in reduced movement and energy expenditure [57]. However, the possible influence of nocturnal predators on daily activity patterns needs to be explored at the current density levels. Many ungulates typically allocate a significant portion of their nighttime hours to rest and rumination [117], although heat stress during dry periods can enhance nocturnal activity [118,119]. Nocturnal activity was low for oryx and gazelle in STNP, except during the summer for oryx. But predators, which usually refrain from hunting during the day because of human activity, might adjust their hunting habits to target prey that is abundant at night, which could increase predation pressure on herbivores exhibiting higher nocturnal activity [120]. The predation risk distribution hypothesis predicts that prey species adjust their activity to minimize their vulnerability during high predation-risk periods [121], by concentrating their activity on low predation-risk situations [122]. Ecological adjustments in activity periods in response to predation pressure can have transformative effects on ecosystems [120]. A pattern of adjustment, in response to nocturnal predators, could account for the bimodal activity patterns of oryx and gazelle we observed in STNP, which align with a strategy of anti-predator behaviour during extended periods of high predation risk, accompanied by intensive activity during periods when predation pressure subsides.

## 5. Conclusions

Our hypothesis that oryx and gazelle differ in at least one dimension of their ecological niche, which contributes to their long-term and stable coexistence in STNP, was supported. While we found similar trends in temporal activity patterns between oryx and gazelle, and a high degree of temporal overlap, the two species show low spatial overlap and differ in parameters accounting for suitable habitat. The primary factors influencing oryx and gazelle habitat choice essentially correspond to grasses and forbs, which they often employ differently. Structural habitat heterogeneity in STNP is a critical determinant of resource and spatial partitioning for oryx and gazelle, resulting in the maintenance of spatial niche separation, a possible mechanism allowing oryx and gazelle to coexist in STNP. Thus, future studies using camera-traps are needed to clarify parameters influencing spatial and temporal niches for coexisting oryx and gazelle, particularly with reference to trophic niches and predation pressures. Finally, it should also be noted that seasonal activity periods may influence the design of effective oryx conservation strategies; as reintroductions occuring during the humid period, particularly between the months of November and January, may be most effective.

## Figures and Tables

**Table 1:** Information-theoretic statistics of seasonal habitat suitability models for oryx in STNP. For each predictor included in the best model, we present AICc, AICc differences (∆AICc), model Akaike weight (wt), selection probability, model-averaged coefficients, and standard error (SE). Predictors are shrubs (SC), forbs (FC), grasses (GC), wadi (WA), altitude (AL), distance to guardhouse or marabout (DP), dirt road (DR), and water troughs (WTs).

Season	Included Predictors								AICc	∆AICc	wt	R^2^
Winter 2020–2021			GC						199.5	0	0.11	0.55
			GC			DP			200.71	1.21	0.06	
			GC					WT	200.92	1.42	0.06	
			GC			DP		WT	201.11	1.61	0.05	
			GC	WA					201.25	1.74	0.05	
			GC		AL				201.29	1.79	0.05	
Selection probability			0.94	0.25	0.25	0.33		0.30				
Coefficient			12.051	−0.004	−0.207	0.003		−0.005				
SE			3.579	0.004	0.198	0.003		0.004				
Winter 2021–2022			GC			DP			203.74	0	0.12	0.51
			GC						204.54	0.79	0.08	
			GC		AL	DP			204.73	0.99	0.08	
			GC					WT	205.69	0.79	0.04	
Selection probability			0.86		0.22	0.49		0.22				
Coefficient			10.430		0.296	0.006		0.006				
SE			3.786		0.263	0.003		0.005				
Spring 2021		FC	GC			DP			209.74	0	0.1	0.58
		FC				DP			210.87	1.13	0.06	
		FC	GC			DP	DR		211.47	1.73	0.04	
Selection probability		0.68	0.54			0.55	0.20					
Coefficient		12.660	7.289			0.008	0.002					
SE		5.875	3.826			0.004	0.002					
Spring 2022		FC	GC				DR	WT	214.78	0	0.12	0.67
		FC	GC			DP	DR	WT	215.3	0.53	0.09	
Selection probability		0.81	0.61			0.31	0.46	0.54				
Coefficient		26.956	17.902			−0.009	0.007	−0.021				
SE		10.929	8.569			0.007	0.004	0.011				
Summer 2021			GC						172.95	0	0.09	0.52
		FC	GC						174.04	0.09	0.05	
	SC	FC	GC						174.08	1.13	0.05	
			GC					WT	174.15	1.2	0.05	
	SC		GC						174.17	1.22	0.05	
			GC				DR		174.76	1.82	0.04	
Selection probability	0.33	0.30	0.86				0.17	0.26				
Coefficient	−0.606	5.639	6.192				−0.001	−0.003				
SE	0.429	3.960	2.251				0.001	0.002				
Summer 2022			GC						173.02	0	0.12	0.49
			GC		AL				173.6	0.58	0.09	
			GC					WT	174.76	1.74	0.05	
Selection probability			0.78		0.31			0.13				
Coefficient			0.109		0.236			−0.003				
SE			4.075		0.166			0.003				
Fall 2021		FC							203.99	0	0.18	0.43
		FC	GC						205.8	1.8	0.07	
Selection probability		0.82	0.23									
Coefficient		15.102	4.865									
SE		4.916	4.585									
Fall 2022		FC		WA					206.38	0	0.1	0.54
		FC					DR		206.69	0.3	0.09	
		FC							207.01	0.62	0.07	
		FC		WA			DR		207.3	0.91	0.06	
		FC				DP	DR		208.31	1.93	0.04	
Selection probability		0.97		0.43		0.20	0.43					
Coefficient		20.425		0.007		−0.002	0.003					
SE		6.102		0.004		0.003	0.002					

**Table 2:** Information-theoretic statistics of seasonal habitat suitability models for gazelle in STNP. For each predictor included in the best model, we present AICc, AICc differences (ΔAICc), model Akaike weight (wt), selection probability, model-averaged coefficients, and standard error (SE). Predictors are shrubs (SC), forbs (FC), grasses (GC), wadi (WA), altitude (AL), distance to guardhouse or marabout (DP), dirt road (DR), and water troughs (WTs).

Season	Included Predictors								AICc	∆AICc	wt	R^2^
Winter 2020–2021		FC	GC				DR		140.18	0	0.12	0.63
	SC	FC	GC				DR		140.42	0.25	0.10	
		FC	GC		AL		DR		141.55	1.38	0.06	
		FC	GC	WA			DR		141.81	1.69	0.06	
Selection probability	0.30	0.83	0.67	0.19	0.40		0.98					
Coefficient	−0.267	3.493	−2.957	0.001	0.10		−0.001					
SE	0.190	1.509	1.38	0.001	0.066		0.000					
Winter 2021–2022		FC		WA	AL				191.93	0	0.06	0.59
	SC	FC						WT	192.02	0.09	0.06	
	SC	FC							192.18	0.24	0.06	
		FC		WA					192.72	0.79	0.04	
	SC	FC					DR		192.86	0.93	0.04	
		FC		WA				WT	193.4	1.47	0.03	
		FC		WA			DR		193.56	1.63	0.03	
		FC		WA	AL		DR		193.74	1.81	0.03	
		FC							193.82	1.89	0.02	
Selection probability	0.4	0.83		0.42	0.3		0.25	0.26				
Coefficient	−1.310	13.272		0.007	0.307		−0.002	−0.005				
SE	0.749	4.723		0.004	0.21		0.002	0.004				
Spring 2021		FC				DP			182.45	0	0.18	0.55
		FC	GC			DP			183.38	0.93	0.11	
		FC							184.08	1.63	0.08	
Selection probability		0.95	0.31			0.67						
Coefficient		10.302	2.829			0.005						
SE		3.383	2.207			0.002						
Spring 2022		FC	GC				DR	WT	209.87	0	0.17	0.68
		FC	GC					WT	210.73	0.85	0.11	
Selection probability		0.85	0.63				0.40	0.80				
Coefficient		25.259	16.269				0.006	−0.023				
SE		9.342	7.489				0.003	0.010				
Summer 2021	SC				AL		DR		189.43	0	0.13	0.52
					AL		DR		190.48	1.06	0.08	
	SC	FC			AL		DR		190.88	1.45	0.06	
Selection probability	0.48	0.17			0.62		0.88					
Coefficient	−1.035	4.704			−0.396		−0.004					
SE	0.595	6.071			0.196		0.001					
Summer 2022				WA		DP			160.21	0	0.13	0.56
			GC	WA		DP			161.02	0.81	0.09	
				WA		DP	DR		161.1	0.89	0.08	
				WA		DP		WT	161.72	1.5	0.06	
	SC			WA		DP			161.84	1.63	0.06	
Selection probability	0.17		0.25	0.88		0.77	0.28	0.19				
Coefficient	0.506		3.852	0.007		−0.004	−0.001	−0.002				
SE	0.495		3.023	0.002		0.001	0.001	0.002				
Fall 2021		FC	GC						170.8	0	0.12	0.56
		FC	GC						171.69	0.89	0.08	
		FC							172.06	1.26	0.06	
		FC	GC		AL				172.55	1.75	0.05	
Selection probability		0.88	0.54		0.26							
Coefficient		8.770	−3.979		0.128							
SE		3.182	2.088		0.118							
Fall 2022		FC		WA					192.05	0	0.07	0.41
		FC							192.33	0.28	0.06	
	SC	FC							192.48	0.43	0.06	
		FC					DR		193.37	1.32	0.04	
		FC				DP			193.74	1.69	0.03	
		FC		WA		DP			194.03	1.76	0.03	
Selection probability	0.18	0.62		0.19		0.17	0.26					
Coefficient	−0.792	11.131		0.004		0.002	0.001					
SE	0.672	4.723		0.003		0.002	0.001					

**Table 3:** Spatial overlap between oryx and gazelle in STNP.

Season	Spatial Overlap
Winter 2020–2021	0.31
Winter 2021–2020	0.35
Spring 2021	0.47
Spring 2022	0.46
Summer 2021	0.38
Summer 2022	0.49
Fall 2021	0.45
Fall 2022	0.57

**Table 4:** The selection ratio *wi* (*n*: number of independent detections) and random use test results of crepuscular, diurnal, and nocturnal time periods for ungulates in STNP.

Antelope Species	*wi* (*n*) in Time Period			ANOVA (df = 2)
	Crepuscular	Diurnal	Nocturnal	
Winter 2020/2021				
Oryx	1.60 (65)	1.16 (96)	0.69 (84)	F = 5.57, *p* = 0.043
Gazelle	1.48 (22)	1.69 (50)	0.38 (17)	F = 8.463, *p* = 0.018
Winter 2021/2022				
Oryx	1.21 (60)	1.13 (114)	0.86 (123)	F = 1.545, *p* = 0.288
Gazelle	1.81 (48)	1.25 (65)	0.56 (41)	F = 13.74, *p* = 0.006
Spring 2021				
Oryx	9.45 (37)	1.04 (109)	0.99 (85)	F = 0.253, *p* = 0.784
Gazelle	1.42 (43)	1.08 (87)	0.72 (46)	F = 3.193, *p* = 0.114
Spring 2022				
Oryx	1.20 (47)	1.13 (107)	0.96 (77)	F = 0.973, *p* = 0.431
Gazelle	1.80 (58)	1.07 (96)	0.57 (42)	F = 16.98, *p* = 0.003
Summer 2021				
Oryx	1.37 (39)	0.79 (69)	1.20 (65)	F = 2.594, *p* = 0.154
Gazelle	1.43 (37)	0.99 (80)	0.78 (46)	F = 4.069, *p* = 0.076
Summer 2022				
Oryx	1.43 (35)	0.63 (46)	1.34 (66)	F = 5.069, *p* = 0.051
Gazelle	2.10 (45)	0.82 (53)	0.71 (31)	F = 47.950, *p* < 0.000
Fall 2021				
Oryx	1.72 (85)	1.14 (132)	0.62 (79)	F = 39.400, *p* < 0.000
Gazelle	1.60 (36)	1.38 (72)	0.46 (27)	F = 4.279, *p* = 0.070
Fall 2022				
Oryx	1.52 (60)	0.95 (91)	0.88 (94)	F = 1.736, *p* = 0.254
Gazelle	1.83 (49)	1.14 (72)	0.56 (45)	F = 13.21, *p* = 0.006

## Data Availability

The data supporting the findings of this study are included within the main document and are available upon reasonable request.

## Supplementary Materials

The following supporting information can be downloaded at: https://www.mdpi.com/article/10.3390/ani14101475/s1, File S1.

## References

[1] El-Beltagy A., Madkour M. (2012). Impact of climate change on arid lands agriculture. Agric. Food Secur..

[2] Gordon I.J., Prins H.H.T. (2019). The Ecology of Browsing and Grazing II.

[3] Lillian S., Redak R.A., Daugherty M.P. (2019). Assessing the role of differential herbivore performance among plant species in associational effects involving the invasive stink bug *Bagrada hilaris* (Hemiptera: Pentatomidae). Community Ecosyst. Ecol..

[4] Ripple W.J., Newsome T.M., Wolf C., Dirzo R., Everatt K.T., Galetti M., Hayward M.W., Kerley G.I.H., Levi T., Lindsey P.A. (2015). Collapse of the world’s largest herbivores. Sci. Adv..

[5] Biggs D., Courchamp F., Martin R., Possingham H.P. (2013). Legal trade of Africa’s rhino horns. Science.

[6] Malcolm J.R., Liu C., Neilson R.P., Hansen L., Hannah L.E.E. (2006). Global warming and extinctions of endemic species from biodiversity hotspots. Conserv. Biol..

[7] Prins H.H., Gordon I.J. (2014). Invasion Biology and Ecological Theory: Insights from a Continent in Transformation.

[8] Schulze E.D., Bouriaud O., Wäldchen N., Eisenhauer H., Walentowski C., Seele E., Helnze U., Pruschitzke G., Dănilă G., Marln D. (2014). Ungulate browsing causes species loss in deciduous forests independent of community dynamics and silvicultural management in central and southeastern europe. Ann. For. Res..

[9] Kasahara M., Fujii S., Tanikawa T., Mori A.S. (2016). Ungulates decelerate litter decomposition by altering litter quality above and below ground. Eur. J. Forest. Res..

[10] IUCN Species Survival Commission (2013). Guidelines for Reintroductions and Other Conservation Translocations.

[11] Newby J.E. (1988). Arid land wildlife in decline: The case of the scimitar-horned oryx. Conserv. Biol. Desert Antelopes..

[12] Hilton-Taylor C. (2000). 2000 IUCN Red List of Threatened Species. IUCN, Gland, Switzerland and Cambridge, UK. https://policycommons.net/artifacts/1372154/2000-iucn-red-list-of-threatened-species/1986326/.

[13] Molcanova R. Update on Scimitar-horned oryx re-introduction in Sidi-Toui National Park, Tunisia. Proceedings of the 7th Annual SSIG Meeting.

[14] IUCN SSC Antelope Specialist Group (2017). *Gazella dorcas*. The IUCN Red List of Threatened Species 2017:E.T8969A50186334.

[15] IUCN SSC Antelope Specialist Group (2023). *Oryx dammah*. The IUCN Red List of Threatened Species 2023:E.T15568A197393805.

[16] Ripple W.J., Estes J.A., Beschta R.L., Wilmers C.C., Ritchie E.G., Hebblewhite M., Berger J., Elmhagen B., Letnic M., Nelson M.P. (2014). Status and ecological effects of the world’s largest carnivores. Science.

[17] Ferretti F., Corazza M., Campana I., Pietrocini V., Brunetti C., Scornavacca D., Lovari S. (2015). Competition between wild herbivores: Reintroduced red deer and Apennine chamois. Behav. Ecol..

[18] Valente A.M., Acevedo P., Figueiredo A.M., Fonseca C., Torres R.T. (2020). Overabundant wild ungulate populations in Europe: Management with consideration of socio-ecological consequences. Mammal. Rev..

[19] Gause G.F. (1934). Experimental analysis of Vito Volterra’s mathematical theory of the struggle for existence. Science.

[20] Wereszczuk A., ZalewskiI A. (2015). Spatial niche segregation of sympatric stone marten and pine marten–avoidance of competition or selection of optimal habitat?. PLoS ONE.

[21] Macarthur R., Levins R. (1967). The limiting similarity, convergence, and divergence of coexisting species. Am. Nat..

[22] Holt R.D., Polis G.A. (1997). A theoretical framework for intraguild predation. Am. Nat..

[23] Grassel S.M., Rachlow J.L., Williams C.J. (2015). Spatial interactions between sympatric carnivores: Asymmetric avoidance of an intraguild predator. Ecol. Evol..

[24] Chesson P. (2000). General theory of competitive coexistence in spatially-varying environments. Theor. Popul. Biol..

[25] Letten A.D., Ke P.J., Fukami T. (2017). Linking modern coexistence theory and contemporary niche theory. Ecol. Monogr..

[26] Carvalho J.C., Cardoso P. (2020). Decomposing the causes for niche differentiation between species using hypervolumes. Front. Ecol. Evol..

[27] Dayan T., Simberloff D. (2005). Ecological and community-wide character displacement: The next generation. Ecol. Lett..

[28] Jonathan D.T., Meiri S., Barraclough T.G., Gittleman J.L. (2007). Species co-existence and character divergence across carnivores. Ecol. Lett..

[29] Hutchinson G.E. (1957). Concluding remarks. Cold Spring Harb. Symp. Quant. Biol..

[30] HilleRisLambers J., Adler P.B., Harpole W.S., Levine J.M., Mayfield M.M. (2012). Rethinking community assembly through the lens of coexistence theory. Annu. Rev. Ecol. Evol. Syst..

[31] Halle S., Stenseth N.C. (2000). Theoretical Considerations—Introduction. Activity Patterns in Small Mammals: An Ecological Approach; with 11 Tables.

[32] Kneitel J.M., Chase J.M. (2004). Trade-offs in community ecology: Linking spatial scales and species coexistence. Ecol. Lett..

[33] Hofmann R.R. (1989). Evolutionary steps of ecophysiological adaptation and diversification of ruminants—A comparative view of their digestive-system. Oecologia.

[34] Murray M.G., Brown D. (1993). Niche separation of grazing ungulates in the Serengeti: An experimental test. J. Anim. Ecol..

[35] Bell R.H.V. (1971). A grazing system in the Serengeti. Sci. Am..

[36] Cromsigt J.P., Prins H.H., Olff H. (2009). Habitat heterogeneity as a driver of ungulate diversity and distribution patterns: Interaction of body mass and digestive strategy. Divers. Distrib..

[37] Azevedo F.C., Lemos F.G., Freitas-Junior M.C., Rocha D.G., Azevedo F.C.C. (2018). Puma activity patterns and temporal overlap with prey in a human-modified landscape at Southeastern Brazil. J. Zool..

[38] Lashley M.A., Cove M.V., Chitwood M.C., Penido G., Gardner B., DePerno C.S., Moorman C.E. (2018). Estimating wildlife activity curves: Comparison of methods and sample size. Sci. Rep..

[39] Kotler B.P., Brown J., Mukherjee S., Berger-Tal O., Bouskila A. (2010). Moonlight avoidance in gerbils reveals a sophisticated interplay among time allocation, vigilance and state-dependent foraging. Proc. R. Soc. B.

[40] Linkie M., Ridout M.S. (2011). Assessing tiger–prey interactions in Sumatran rainforests. J. Zool..

[41] Ramesh T., Kalle R., Sankar K., Qureshi Q. (2012). Spatio-temporal partitioning among large carnivores in relation to major prey species in W estern G hats. J. Zool..

[42] Johnsingh A.J.T. (1983). Large mammal prey–predators in Bandipur. J. Bombay Nat. Hist. Soc..

[43] Bowland A.E., Perrin M.R. (1995). Temporal and spatial patterns in blue duikers Philatomba monticola and red duikers Cephalophus natalensis. J. Zool..

[44] Nathan R., Spiegel O., Fortmann-Roe S., Harel R., Wikelski M., Getz W.M. (2012). Using tri-axial acceleration data to identify behavioral modes of free-ranging animals: General concepts and tools illustrated for griffon vultures. J. Exp. Biol..

[45] Mori E., Andreoni A., Cecere F., Magi M., Lazzeri L. (2020). Patterns of activity rhythms of invasive coypus Myocastor coypus inferred through camera-trapping. Mamm. Biol..

[46] Viviano A., Mori E., Fattorini N., Mazza G., Lazzeri L., Panichi A., Strianese L., Mohamed W.F. (2021). Spatiotemporal overlap between the European brown hare and its potential predators and competitors. Animals.

[47] Li X., Tian H., Piao Z., Wang G., Xiao Z., Sun Y., Gao E., Holyoak M. (2022). cameratrapR: An R package for estimating animal density using camera trapping data. Ecol. Inform..

[48] Agha M., Batter T., Bolas E.C., Collins A.C., Gomes da Rocha D., Monteza-Moreno C.M., Preckler-Quisquater S., Sollmann R. (2018). A review of wildlife camera trapping trends across Africa. Afr. J. Ecol..

[49] Greenberg S., Godin T., Whittington J. (2019). Design patterns for wildlife-related camera trap image analysis. Ecol. Evol..

[50] Harmsen B.J., Foster R.J., Doncaster C.P. (2011). Heterogeneous capture rates in low density populations and consequences for capture-recapture analysis of camera-trap data. Popul. Ecol..

[51] Pfeffer S.E., Spitzer R., Allen A.M., Hofmeester T.R., Ericsson G., Widemo F., Singh N.J., Cromsigt J.P. (2018). Pictures or pellets? Comparing camera trapping and dung counts as methods for estimating population densities of ungulates. Remote Sens. Ecol. Conserv..

[52] Mori E., Bagnato S., Serroni P., Sangiuliano A., Rotondaro F., Marchianò V., Cascini V., Poerio L., Ferretti F. (2019). Spatiotemporal mechanisms of coexistence in an European mammal community in a protected area of southern Italy. J. Zool..

[53] Houngbégnon F.G., Cornelis D., Vermeulen C., Sonké B., Ntie S., Fayolle A., Fonteyn D., Lhoest S., Evrard Q., Yapi F. (2020). Daily activity patterns and co-occurrence of duikers revealed by an intensive camera trap survey across central african rainforests. Animals.

[54] You Z., Lu B., Du B., Liu W., Jiang Y., Ruan G., Yang N. (2022). Spatio-Temporal Niche of Sympatric Tufted Deer (*Elaphodus Cephalophus*) and Sambar (*Rusa unicolor*) Based on Camera Traps in the Gongga Mountain National Nature Reserve, China. Animals.

[55] Meliane M.K., Petretto M., Saidi A., Chetoui A., Gilbert T., Nasri-Ammar K. (2023). Daily and seasonal activity patterns of the dorcas gazelle, scimitar-horned oryx, north-African ostrich and canids in an arid habitat. Afr. J. Ecol..

[56] Karanth K.K. (2016). Wildlife in the matrix: Spatio-temporal patterns of herbivore occurrence in Karnataka, India. Environ. Manag..

[57] Li S., McShea W.J., Wang D., Shen X., Bu H., Guan T., Wang F., Gu X., Zhang X., Liao H. (2020). Construction progress of the Camera-trapping Network for the Mountains of Southwest China. Biodivers. Sci..

[58] Karem A., Ksantini M., Schoenenberger A., Waibel T. (1993). Contribution à la Régénération de la Végétation dans le Parcs Nationaux en Tunisie Aride.

[59] Tarhouni M., Ben Hmida W., Neffati M. (2017). Long-term changes in plant life forms as a consequence of grazing exclusion under and climatic conditions. Land Degrad. Dev..

[60] Lyra-Jorge M.C., Ribeiro M.C., Ciocheti G., Tambosi L.R., Pivello V.R. (2010). Influence of multi-scale landscape structure on the occurrence of carnivorous mammals in a human-modified savanna, Brazil. Eur. J. Wildl. Res..

[61] Meliane M.K., Saidi A., Petretto M., Nasri-Ammar K., Taghouti E., Guidara H., Boufaroua M., Woodfine T., Gilbert T. (2023). African houbara (*Chlamydotis undulata undulata*) confirmed in Sidi Toui National Park, Tunisia. Afr. J. Ecol..

[62] Meliane M.K., Saidi A., Petretto M., Woodfine T., Riordan P., Gilbert T., Taghouti E., Guidara H. (2023). The crested porcupine in Tunisia’s semi-arid steppes. Oryx.

[63] Kalan A.K., Hohmann G., Arandjelovic M., Boesch C., McCarthy M.S., Agbor A., Angedakin S., Bailey E., Balongelwa C.W., Bessone M. (2019). Novelty response of wild African apes to camera traps. Curr. Biol..

[64] Bargaoui Z., Tramblay Y., Lawin E.A., Servat E. (2014). Seasonal precipitation variability in regional climate simulations over Northern basins of Tunisia. Int. J. Climatol..

[65] Ridout M.S., Linkie M. (2009). Estimating overlap of daily activity patterns from camera trap data. J. Agric. Biol. Environ. Stat..

[66] Jiménez J., Nuñez-Arjona J.C., Rueda C., González L.M., García-Domínguez F., Muñoz-Igualada J., López-Bao J.V. (2017). Estimating carnivore community structures. Sci. Rep..

[67] Li J., Xue Y., Zhang Y., Dong W., Shan G., Sun R., Hacker C., Wu B., Li D. (2020). Spatial and temporal activity patterns of Golden takin (*Budorcas taxicolor bedfordi*) recorded by camera trapping. PeerJ.

[68] Rosenzweig M.L. (1981). A thoery of habitat selection. Ecology.

[69] Bergeson S.M., Carter T.C., Whitby M.D. (2013). Partitioning of foraging resources between sympatric Indiana and little brown bats. J. Mammal..

[70] Daget P., Poissonet J. (1971). Une méthode d’analyse phytologique des prairies. Critères d’application. Ann. Agron..

[71] He F., Legendre P. (2002). Species diversity patterns derived from species–area models. Ecology.

[72] He F., Legendre P. (1996). On species-area relations. Am. Nat..

[73] Blake J.G., Mosquera D., Guerra J., Loiselle B.A., Romo D., Swing K. (2011). Mineral licks as diversity hotspots in lowland forest of eastern Ecuador. Diversity.

[74] Bowkett A.E., Rovero F., Marshall A.R. (2008). The use of camera-trap data to model habitat use by antelope species in the Udzungwa Mountain forests, Tanzania. Afr. J. Ecol..

[75] McCullagh P. (2019). Generalized Linear Models.

[76] Mary S.W., Antoine G. (2009). Do pseudo-absence selection strategies influence species distribution models and their predictions? An information-theoretic approach based on simulated data. BMC Ecol..

[77] Burnham K.P., Anderson D.R. (2006). Model Selection and Inference: A Practical Information-Theoretic Approach.

[78] Whittingham M.J., Stephens P.A., Bradbury R.B., Freckleton R.P. (2006). Why do we still use stepwise modelling in ecology and behaviour?. J. Anim. Ecol..

[79] Pianka E.R. (1973). The structure of lizard communities. Annu. Rev. Ecol. Syst..

[80] Bennie J.J., Duffy J.P., Inger R., Gaston K.J. (2014). Biogeography of time partitioning in mammals. Proc. Natl. Acad. Sci. USA.

[81] de Satgé J., Teichman K., Cristescu B. (2017). Competition and coexistence in a small carnivore guild. Oecologia.

[82] Mella-Méndez I., Flores-Peredo R., Pérez-Torres J., Hernández-González S., González-Uribe D.U., del Socorro Bolívar-Cimé B. (2019). Activity patterns and temporal niche partitioning of dogs and medium-sized wild mammals in urban parks of Xalapa, Mexico. Urban Ecosyst..

[83] Manly B.F.J., McDonald L.L., Thomas D.L., McDonald T.L., Erickson W.P. (2002). Resource Selection by Animals: Statistical Design and Analysis for Field Studies.

[84] Lynam A.J., Jenks K.E., Tantipisanuh N., Chutipong W., Ngoprasert D., Gale G.A., Steinmetz R., Sukmasuang R., Bhumpakphan N., Lon I. (2013). Terrestrial activity patterns of wild cats from camera-trapping. Raffles Bull. Zool..

[85] Meredith M., Ridout M. (2014). Overlap: Estimates of Coefficient of Overlapping for Animal Activity Patterns. https://cran.r-project.org/package=overlap.

[86] Monterroso P., Alves P.C., Ferreras P. (2014). Plasticity in circadian activity patterns of mesocarnivores in Southwestern Europe: Implications for species coexistence. Behav. Ecol. Sociobiol..

[87] R Core Team (2023). R: A Language and Environment for Statistical Computing.

[88] Meliane M.K., Saidi A., Petretto M., Gilbert T., Nasri-Ammar K. (2023). Temporal and spatial distribution of dorcas and slender-horned gazelles in a Saharan habitat. J. Wildl. Manag..

[89] Cooke R.S.C., Woodfine T., Petretto M., Ezard T.H.G. (2016). Resource partitioning between ungulate populations in arid environments. Ecol. Evol..

[90] Walther F.R. (1969). Flight behaviour and avoidance of predators in Thomson’s gazelle (*Gazella thomsoni* Guenther 1884). Behaviour.

[91] Costelloe B.R., Rubenstein D.I. (2018). Temporal structuring of vigilance behaviour by female Thomson’s gazelles with hidden fawns. Anim. Behav..

[92] Abáigar T., Cano M., Djigo C.A., Gomis J., Sarr T., Youm B., Fernández-Bellon H., Ensenyat C. (2016). Social organization and demography of reintroduced Dorcas gazelle (*Gazella dorcas neglecta*) in North Ferlo Fauna Reserve. Senegal. Mamm..

[93] Abáigar T., Cano M., Ensenyat C. (2018). Time allocation and patterns of activity of the dorcas gazelle (*Gazella dorcas*) in a sahelian habitat. Mamm. Res..

[94] Newby J.E. (1974). The ecological resources of the Ouadi Rimé-Ouadi Achim faunal reserve. Arada, UNDP/FA Wildlife Conservation and Management Project CHD/69/004.

[95] Hofmann R.R., Stewart D.R.M. (1972). Grazer or browser: A classification based on the stomach-structure and feeding habits of East African ruminants. Mammalia.

[96] Hofmann R.R. (1973). Ruminant Stomach: Stomach Structure and Feeding Habits of East African Game Ruminants.

[97] Schmidt-Nielsen K. (1984). Scaling: Why Is Animal Size so Important?.

[98] Haskell J.P., Ritchie M.E., Olff H. (2002). Fractal geometry predicts varying body size scaling relationships for mammal and bird home ranges. Nature.

[99] Henley S.R., Ward D., Schmidt I. (2007). Habitat selection by two desert-adapted ungulates. J. Arid. Environ..

[100] Dragesco-Joffé A. (1993). La vie sauvage au Sahara. Lausanne. Rev. Ecol..

[101] Robinson S.E., Weckerly F.W. (2010). Grouping patterns and selection of forage by the scimitar-horned oryx (*Oryx dammah*) in the Llano Uplift region of Texas. Southwest. Nat..

[102] Wacher T. (1988). Social organisation and ranging behaviour in the Hippotraginae. Conserv. Biol. Desert Antelopes..

[103] Spalton J.A. (1999). The food supply of Arabian oryx (*Oryx leucoryx*) in the desert of Oman. J. Zool..

[104] Pahkala K., Pihala M. (2000). Different plant parts as raw material for fuel and pulp production. Ind. Crop. Prod..

[105] Jaballah S., Gribaa A., Volaire F., Ferchichi A. (2008). Ecophysiological Reponses of perennial grasses Stipa lagascae and Dactylis glomerata under soil water deficit. Opt. Mediterr. Ser. A.

[106] Du Toit J.T., Olff H. (2014). Generalities in grazing and browsing ecology: Using across-guild comparisons to control contingencies. Oecologia.

[107] Bukombe J., Kittle A., Senzota R.B., Kija H., Mduma S., Fryxell J.M., Magige F., Mligo C., Sinclair A.R.E. (2019). The influence of food availability, quality and body size on patch selection of coexisting grazer ungulates in western Serengeti National Park. Wildl. Res..

[108] Schaller G.B. (1998). Mongolia’s golden horde, a million migrating gazelles. Wildl. Conserv..

[109] Iason G.R., Van Wieren S.E. (1999). Digestive and ingestive adaptations of mammalian herbivores to low quality forage. Herbivores: Between Plants and Herbivores.

[110] Breuil M., Mayeur J.P., Thille F. (1998). Kenya-Tanzanie: Le Guide du Safari, Faune et Parcs.

[111] Massé A., Côté S. (2013). Spatiotemporal variations in resources affect activity and movement patterns of white-tailed deer (*Odocoileus virginianus*) at high density. Can. J. Zool..

[112] Hetem R.S., Strauss W.M., Fick L.G., Maloney S.K., Meyer L.C.R., Shobrak M., Fuller A., Mitchell D. (2012). Does size matter? Comparison of body temperature and activity of free-living Arabian oryx (*Oryx leucoryx*) and the smaller Arabian sand gazelle (*Gazella subgutturosa marica*) in the Saudi desert. J. Comp. Physiol. B.

[113] Babor H., Okab A.B., Samara E.M., Abdoun K.A., Omar A.T., Al-Haidary A.A. (2014). Adaptive thermophysiological adjustments of gazelles to survive hot summer conditions. Pak. J. Zool..

[114] Espinosa S., Salvador J. (2017). Hunters landscape accessibility and daily activity of ungulates in Yasuní Biosphere Reserve, Ecuador. Therya.

[115] Noor A., Mir Z.R., Veeraswami G.G., Habib B. (2017). Activity patterns and spatial co-occurrence of sympatric mammals in the moist temperate forest of the Kashmir Himalaya, India. Folia Zool..

[116] de Sá Alves L.C.P., Andriolo A. (2005). Camera traps used on the mastofaunal survey of Araras Biological Reserve, IEF-RJ. Rev. Bras. Zoociências..

[117] Leuthold W. (1977). African Ungulates: A Comparative Review of their Ethology and Behavioural Ecdogy.

[118] Merrill E.H. (1991). Thermal constraints on use of cover types and activity time of elk. Appl. Anim. Behav. Sci..

[119] Klassen N.A., Rea R.V. (2008). What do we know about nocturnal activity of moose?. Alces.

[120] Gaynor K.M., Hojnowski C.E., Carter N.H., Brashares J.S. (2018). The influence of human disturbance on wildlife nocturnality. Science.

[121] Lima S.L., Bednekoff P.A. (1999). Temporal variation in danger drives antipredator behavior: The predation risk allocation hypothesis. Am. Nat..

[122] Fenn M.G., Macdonald D.W. (1995). Use of middens by red foxes: Risk reverses rhythms of rats. J. Mammal..

